# Investigation of health benefits of cocoa in human colorectal cancer cell line, HT-29 through interactome analysis 

**Published:** 2019

**Authors:** Mona Zamanian-Azodi, Mostafa Rezaei-Tavirani

**Affiliations:** 1 *Student Research Committee, Proteomics Research Center, Shahid Beheshti University of Medical Sciences, Tehran, Iran*; 2 *Proteomics Research Center, Faculty of Paramedical Sciences, Shahid Beheshti University of Medical Sciences, Tehran, Iran*

**Keywords:** Colon cancer, Cocoa extraction, Transcriptome, Interactome

## Abstract

**Aim:**

This bioinformatics study aims to identify the potential key genes influenced by cocoa extraction treatment on colon cancer cell line HT-29 after 24h.

**Background:**

Cocoa consumption has been claimed to have beneficial effects on human body including protection against diseases such as different types of cancers. However, the mechanisms behind this is still remained to be studied.

**Methods:**

The microarray dataset (GSE94154) from GEO, was the source for differentially expressed genes (DEGs) extraction through GEO2R analysis. The comparison was between 3 controls of colorectal cell line HT-29 and 3 ones incubated with 500 µg cocoa extraction after 24 h. Afterwards, the top significant DEGs were assigned for protein-protein interaction network construction and analysis by Cytoscape v 3.7.0. and the related applications.

**Results:**

The findings indicate that there are 222 up-regulated and 28 down-regulated genes among 250 top-ranked DEGs in cocoa incubated group. What is more, centrality analysis of the DEGs network identified 10 hub-bottlenecks that ISG15, MX 1, and STAT1 were among the significant differential expression genes with the contribution in type 1 interferon signaling pathway, positive regulation of erythrocyte differentiation, and negative regulation of viral genome replication.

**Conclusion:**

In conclusion, the underlying mechanisms of cocoa treatment could be clarified by its up-regulatory and modulatory effect on prominent genes of tumor suppressor family. In other words, valuable clues for future clinical studies of cocoa health benefits are highlighted as anticancer agent in this study once validation studies are carried out.

## Introduction

 Cancer of colon (CRC) as the third frequent type of cancer globally is also accounted for the second cause of cancer-associated death in western world (1, 2). The lack of treatment approaches with higher efficiency and lower side effects for this type of cancer encourage alternatives with regards to plant-based medicines (3, 4). It is well-documented by many studies that, there are promising health benefits of phenolic plants such as cocoa powder on human body (3, 5). Historically, from seventeenth century, these substances has been applied as food and medicine for many types of disease, especially in Europe (5). Of which, cardio and neuro protection, metabolism regulation, and cancer prevention are the well-known properties of this popular seed (6, 7). In diseases such as cancer that oxidative stress is the related process, cocoa extractions showed effective (5). The potential beneficial effect of cocoa intake is due to bioactive phytochemical agents that could alter the molecular profiles such as genome, proteome, and metabolome in human body (6, 8). Some evidence representing positive effects of cocoa on colonic cancer are existing that suggests chemo-preventive values of cocoa ingredients (9). In addition, breast cancer study showed that cocoa could inhibit the growth of malignant cells. In a way that, down-regulation or dephosphorylation of many regulatory proteins contributing in cell cycle are linked to pentameric procyanidin cytotoxic influence (10). Cocoa plays a protective role against abnormal growth, mutagen, toxicity, and carcinogens (5). As earlier indicated, molecular profile alteration can be induced by the intake of cocoa. Gene expression analysis as one of the high-throughput evaluations shows the molecular pattern changes in the presence of any possible treatments (11). Furthermore, bioinformatics add more information to genomic studies by identification of the most promising differentially expressed genes. One of these ways is by protein-protein interaction network mapping of these genes. Therefore, this in-silico evaluation seeks to underpin the underlying mechanisms by which cocoa extraction effects on colon cancer transcriptome. 

## Methods

In this bioinformatical study, expression profiling by array data was obtained from the open access database, Gene Expression Omnibus (GEO). The study that was designated for our evaluation is from 6 samples including groups of 3 control (colorectal adenocarcinoma cells) and 3 treated ones with cocoa extraction (500 µg). Due to dose dependency of cocoa effects, appropriate dose of cocoa extract is selected. The study identifies with Series Accession: GSE94154 and Platform: GPL570 that was conducted by Ciudad CJ, et al. in 2018 entitled, “Gene expression after 24h Cocoa Extract incubation in HT29 cells (Homo sapiens)”. An online analyzer GEO2R, based on the R programming language (https://www.ncbi.nlm.nih.gov/ geo/geo2r/) in GEO was used to identify differentially expressed genes (DEGs) between two groups of study. In GEO2R, at first data were preprocessed via box plot analysis and then the DEGs were analyzed by the application of t-test, and the top ranked genes (250 significant ones) were screened and assigned by the consideration of adj. P-value via Benjamini and Hochberg correction method. The adjusted p-value was set to ≤ 0.001 and fold change equal and more than 1.5 as cut offs for DEGs that were then followed for interaction network analysis. In the Cytoscape v.3.7.0, an open source for network construction (12), DEGs network was expanded with the addition of 50 neighbor genes and visualized as a protein-protein interaction network via String db source 1.4.0 (protein query) (13). In a way that, a network was imported by considering confidence score cut off ≥0.5. For centrality analysis, Network Analyzer investigated two important criteria, degree and betweenness centrality (14). Nodes (genes) with highest degree possess large amount of connectivity while nodes with high betweenness centrality are those with great impact on a path connecting other elements of a PPI network (15). Furthermore, to check the expression pattern and significance of hub-bottlenecks, CluePedia v1.5.3 was used to assess different spots of each hub-bottlenecks expression profile. ClueGO v 2.5.3+ CluePedia v1.5.3 as a Cytoscape tools provide gene, protein, and miRNA annotations in terms of gene ontology and pathway analysis (16). Enrichments for gene ontology can be as biological process (BP), cellular component (CC) and molecular function (MF). The statistical criteria for this analysis is as follow: GO term min number of genes=2 and min gene percentage in term=3. Moreover, p-value correction method is Bonfferoni step down as the default option. The enrichment/depletion: two-sided hypergeomtric test. GO term network connectivity was also set to 0.5 as the medium range. GO tree interval min level as 3 and max level as 8. P-value was also set to ≤ 0.05. 

## Results

Value distribution of samples are graphically shown via box-plotting and compared for evaluating the quality of which as a requirement for continuing the analysis. A network of differentially expressed genes was constructed via String database integrated in Cytoscape. This network consists of DEGs that were with names and significant with adj. P ≤ 0.001 and fold change≥ 1.5. GEO2R analysis showed 51736 expressed genes that among the 250 top significantly expressed genes, 222 of them were up-regulated while 28 ones were down-regulated. Of which, 237 ones were identified in String db and constructed with the addition of 50 genes. Together, 287 genes and 4481 edges were the elements of this protein-protein interaction network. For the centrality analysis, at first, a sub-network of the PPI network was created with 242 nodes and 4477 interactions. This sub-network was then applied for the network centrality analysis via Network Analyzer (the data are not shown). Centrality analysis via determination of degree and betweenness was conducted by setting the cut off to 10% top ranked ones. The common top ranked of these two criteria values were then considered as hub-bottlenecks. The hub-bottlenecks are the most highlighted central genes that could be valuable for our further analysis in this study (table 1). 

**Table 1:** The list of hub-bottlenecks of network of colon cancer cell line HT-29 treated with cocoa extraction. BC refers to betweenness centrality

**R**	**Display name**	**Description**	**Degree**	**BC**	**Query term**
1	ISG15	ISG15 ubiquitin-like modifier	111	0.02	ISG15
2	STAT1	signal transducer and activator of transcription 1, 91kDa	105	0.03	STAT1
3	MX1	myxovirus (influenza virus) resistance 1, interferon-inducible protein p78 (mouse)	95	0.02	MX1
4	TP53	tumor protein p53	89	0.04	-
5	GAPDH	glyceraldehyde-3-phosphate dehydrogenase	87	0.02	-
6	PRDM10	PR domain containing 10	85	0.03	-
7	EGR1	early growth response 1	80	0.02	-
8	IL6	interleukin 6 (interferon, beta 2)	78	0.02	-
9	AKT1	v-akt murine thymoma viral oncogene homolog 1	76	0.04	-
10	TNF	tumor necrosis factor	76	0.02	-

**Table 2:** Abbreviations S-DE-HB, FC, FDR, and NS indicate significant differentially expressed hub-bottlenecks, fold change, false discovery rate, and number of spots, respectively

**Rank**	**S-DE-HB**	**Protein name**	**Expression Condition**	**FC**	**FDR**	**NS**
12	MX1	MX dynamin like GTPase 1	Up	4.5	0.00007	1
211	ISG15	ISG15 ubiquitin-like modifier	Up	2.3	0.0008	1
38	STAT1	signal transducer and activator of transcription 1	UP	2.3	0.00014	7

**Figure 1:**
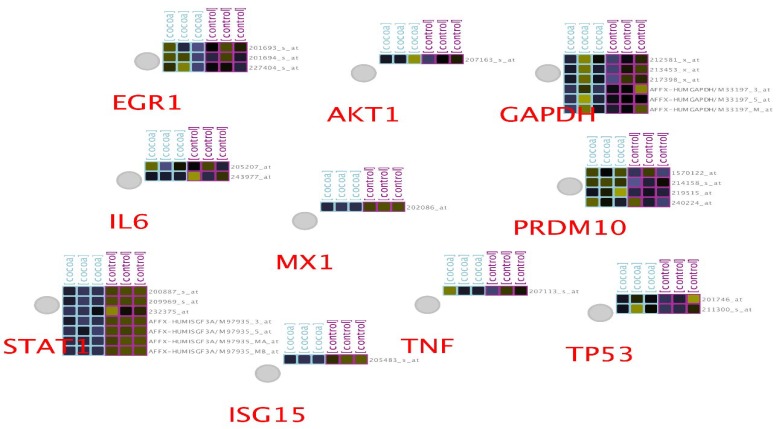
Normalized expression data for 10 hub-bottlenecks shown as label nodes from the merged file in CluePedia. Blue and purple color indicate groups of samples which are 3 controls and 3 treated. Different color pattern range implies on maximum positive expression changes to negative expression (purple to green)

To gain a more depth understanding of DEGs hub-bottlenecks, the expression pattern of them were visualized and assessed via CluePedia. Some genes correspond to more than one spot in the expression profile of colon cancer (figure 1). 

MX1, TNF, AKT1, and ISG15 have only one spot in our sample while others express more than one. There is no missing expression values for the explored genes in the expression profile file. That is, all the query genes in our sample are expressed. EGR1 genes consists of three spots that none of them including 201693_s_at, 227404_s_at, and 201694_s_at show any statistical significant differential expression in the treated group. The rank for these genes are 25669, 36458, and 42457, respectively (ranking is based on statistically significance). IL6 shows two spots, 205207 and 243977_at that none were significant DEGS. AKT1 and TNF with only one spot available in the expression file (207163_s_at) and 207113_s_at ranked as 33249 and 48822 neither were significant nor as differential expressed genes. GAPDH, the next hub-bottleneck has 6 spots here that none of which reflect significant differential expression. 43553, 46702, 52403, 51266, 51383, 52366 are the ranks of these spots (M33197_3_at, 212581_x_at, 213453_x_at, M33197_M_at, M33197_5_at, and 217398_x_at), in which showing highest and lowest levels of significance and fold change, respectively. PRDM10 the next gene, contains 4 spots (219515_at, 214158_s_at, 214158_s_at, and 240224_at here that they ranking are 14479, 25033, 25033, and 33667 that none of which were met the criteria for S-DEGs. 

**Figure 2:**
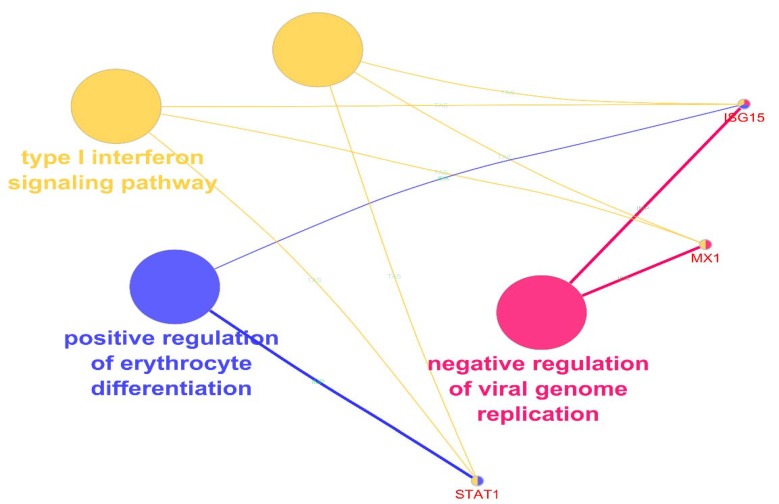
The biological role of S-DE-HB in three groups including type 1 interferon signaling pathway, positive regulation of erythrocyte differentiation, and negative regulation of viral genome replication in yellow, blue, and pink, respectively. These groups are showed in ClueGO+CluePedia panel as fused terms. GO terms are fused. p≤ 0.05

Finally, TP53 as the last exploring hub-bottleneck with following characteristics with the ranks of (18357 and 50817) and IDs (211300_s_at and 201746_at) did not reached the desirable threshold for differential expression significance outcome. ISG15, MX1, and STAT1 are from the top 250 significant DEGs that among them the first two show only one spot from the experience, which are present in the highest level of significance. STAT1, on the other hand, has seven spots in the experiment that all of them except (AFFX-HUMISGF3A/M97935_5_at) are present among the top 250 significant DEGs. All of these genes are up-regulated in the treated group. In addition, STAT1 possess the highest number of spots in this experience. Additionally, enrichment analysis of the DEGs in terms of biological process identifications was performed, followed by expression network construction as shown in figure 2.

## Discussion

Colon cancer genes affected in their expression by cocoa extraction are studied to get a more resolution of health benefits of this food. Besides, complementary studies of high-throughput investigations such as bioinformatics, aid more understanding of molecular profile alterations (17). In this approach, we pursuit understanding the most influenced central candidates of colon cell line, HT-29 in the treatment of cocoa extraction. For this aim, cross-comparison of expression profile of sample groups of control and treated validated the further analysis of which for DEGs profiling as shown in supplement figure 1. The obtained top 250 identified DEGs were then considered for network construction and analysis, in which 10 prominent nodes were then introduced as tabulated in table 1. Among them, MX1, ISG15, and STAT1 are from top significant differentially expressed genes in the treated category. The other hub-bottlenecks are from addition neighbors around our query genes. In addition, these three hub-bottlenecks possess the highest values of degree in the hub-bottleneck rankings. MX1 and ISG15 have the highest degree whereas TP 53 and AKT1 are the highest in betweenness centrality values. A better insight of these three identified and the rest of hub-bottlenecks could be gained by exploring them in expression profile of the studied samples. CluePedia could assist this matter by linking the expression profile of any query genes via GEO database. The examination represented the expression profile of the 10 hub-bottlenecks as shown in figure 1 that there are two types of genes. Those with only one spot and others with many spots. The overall analysis of genes with many spots showed that only STAT1 is statistically significant in differential expression of treated subjects. Furthermore, MX1 and ISG15 from genes with one spot group are significantly differential in expression. Therefore, MX1, ISG15, and STAT1 are the only significant differential expressed hub-bottlenecks in treatment with cocoa. These three genes are focused to see what their behavior is in our samples. The important thing about these three genes is that they are over-expressed in exposure to cocoa. Moreover, expression profile analysis of these genes show that MX1 and STAT1 are the most significantly expressed hub-bottlenecks in the treated subjects as mentioned in table 2. To see what the role of these S-DE hub-bottlenecks could be in the mechanisms of cocoa treatment, enrichment analysis of them were carried out. The functionally grouped terms as a network are presented in figure 2 in which express that our significant differentially expressed genes are integrated in three different biological process. It can be inferred that including type 1 interferon signaling pathway, positive regulation of erythrocyte differentiation, and negative regulation of viral genome replication processes could be effected by cocoa extraction treatment. The type 1 interferon signaling pathway is the most highlighted process in this study. All these three genes are active in interferon pathway; likewise, previously it was also indicated by the main study that 48 interferons were altered in HT-29 cocoa incubated. It shows that the interferon pathway could be one of the centers of modulatory function of cocoa exposure with highlighting contribution of these three genes. These genes were also searched against literature and their possible contribution in cancer was reviewed. MX1 or MXA (myxovirus resistance protein 1) is recognized as antiviral fighter from innate immune system. In addition to this, this protein is known to be a tumor mutation inhibitor that its mutation is significant in many different types of cancers including colorectal tumorigenesis (18, 19). The next genes is ISG15 (Interferon-stimulated gene 15) another interferon, that is also from innate immune system and it is active in tumor inhibition (20). There are many studies claiming that this protein is an effective inhibitor on different forms of cancer progression (21). The low level of this interferon protein could promote the tumor progression (22). The last important genes from our investigation is the first member of STAT family named STAT1 (signal transducer and activator of transcription 1). This gene is similarly involved in interferon pathway and activated in responding to IFNs stimulations. The prominent assistance of this gene in cancer suppression has been well-established. Nevertheless, it should be noted that, its contradictory mysterious role in different cancer types is also suggested by some studies (23). All these genes are parts of interferon signaling pathway of digestive system malignancies (22). In this study, it is detected that they are up-regulated in the exposure with cocoa extract. This fact can suggest that cocoa is possibly beneficial in the trigger of tumor suppressing functions in cancers such as colon. On the other hand, one of the cancer cure approaches is by immunotherapy that has received a great deal of supporting evidence. In this respect, IFN-alpha agents are enormously applied as clinical interventions via corresponding mechanisms in human body. One of which is by modulation and up-regulation of tumor suppressors to halt proliferation and progression of many cancer types (24). Surprisingly, it can be concluded that, cocoa could act as a natural food supply that can facilitate anticancer signaling in human body by the molecular modulations. In other words, majority of cocoa consumption and consequently modulation could be on the essential tumor suppressor genes that are the fundamental elements of the interferon pathway. Since incubation of colon cancer cell line with cocoa extract was accompanied with tumor suppressor genes ’up-regulation, cocoa could be considered as a cancer preventive agent.

The therapeutic benefits of cocoa consumption and colon cancer risk could be featured with regulatory modulations of ISG15, MX1, and STAT as the central tumor suppressor genes in the protein-protein interaction network. Therefore, the anticancer properties of cocoa is suggested by this study; however, validation tests are encouraged in this regard.
